# 168. Syndrome-Based Analysis of Oral Antimicrobial Stewardship Opportunities at Hospital Discharge

**DOI:** 10.1093/ofid/ofab466.370

**Published:** 2021-12-04

**Authors:** Jessica Cunningham, Shawn Binkley, Tanya Uritsky, Stephen Saw, Sonal Patel, Tiffany Lee, Keith W Hamilton, Kathleen Degnan, Lauren Dutcher, Vasilios Athans

**Affiliations:** Hospital of the University of Pennsylvania, Philadelphia, Pennsylvania

## Abstract

**Background:**

Suboptimal oral antibiotic prescriptions (OAPs) are prevalent at discharge and contribute to treatment failure, resistance, toxicity, and excess costs. Syndrome-specific prescribing patterns have not been widely described at discharge, nor have specific reasons for excessive treatment durations (the most commonly cited prescribing error).

**Methods:**

Retrospective cohort of patients discharged from a general medicine service at an academic hospital with ≥1 OAP for urinary tract infection (UTI), skin and soft tissue infection (SSTI), or lower respiratory tract infection (LRTI). Study period varied to include a random sample of encounters occurring after the most recent institutional guideline update for each syndrome. Exclusions: multiple infectious indications, discharge against medical advice, parenteral antibiotics at discharge, pregnancy, cystic fibrosis, and immunocompromising conditions. Discharge OAPs were assessed for suboptimal selection, dose, frequency, or duration according to institutional guidelines (with secondary adjudication).

**Results:**

Analysis included 160 encounters: 70 UTIs, 66 SSTIs, and 24 LRTIs. Of 71 (44%) culture-positive infections, Enterobacterales (61%) and *Streptococcus* spp. (15%) were most often identified. In total, 180 OAPs were issued – most commonly cefpodoxime (21%), cefadroxil (18%), and doxycycline (17%). Overall, 99 (62%) encounters were associated with a suboptimal discharge OAP. Of 138 suboptimal characteristics identified, suboptimal duration was most frequent (57%), specifically excessive duration (45%). Proportion of suboptimal OAPs and their underlying reasons are analyzed by syndrome in Figures 1 and 2, respectively. Miscalculation (39%), intentional selection of guideline-discordant duration (29%), and omission of inpatient antibiotic days (19%) were the most frequent reasons for suboptimal duration (Fig. 3).

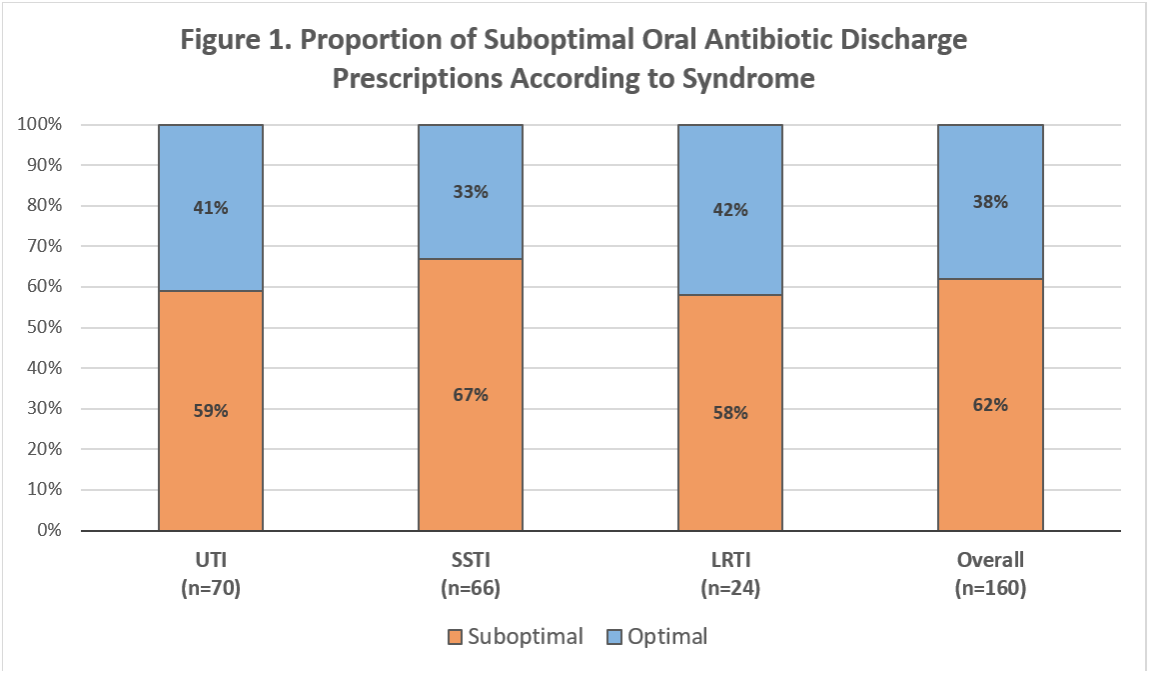

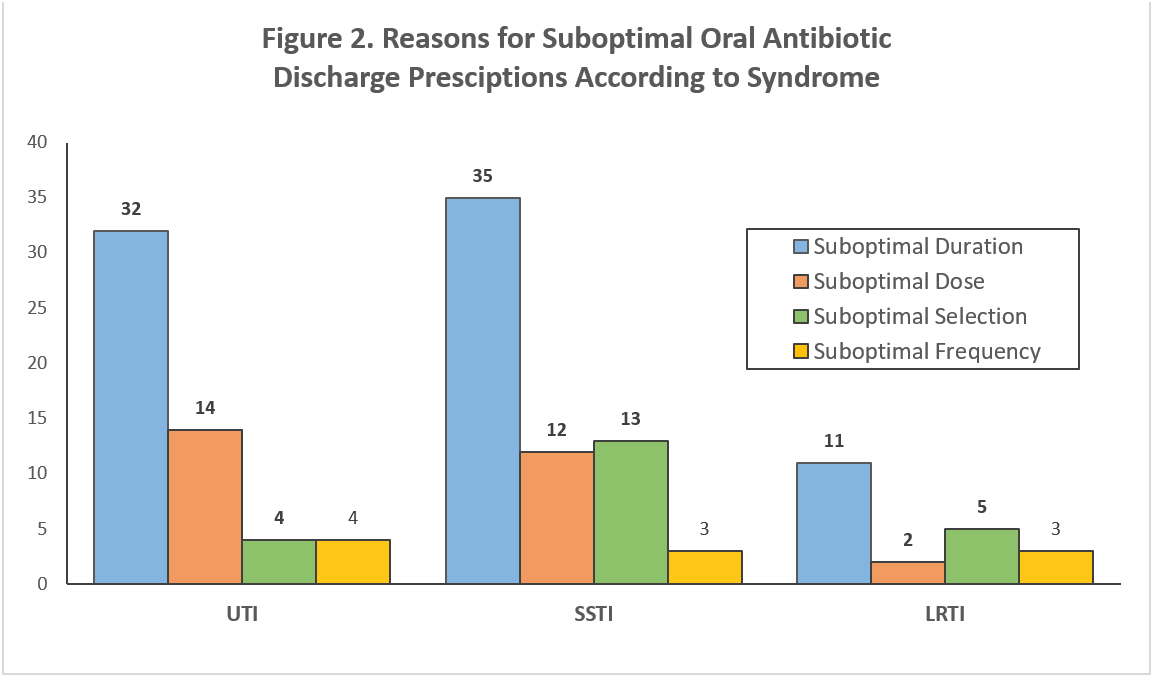

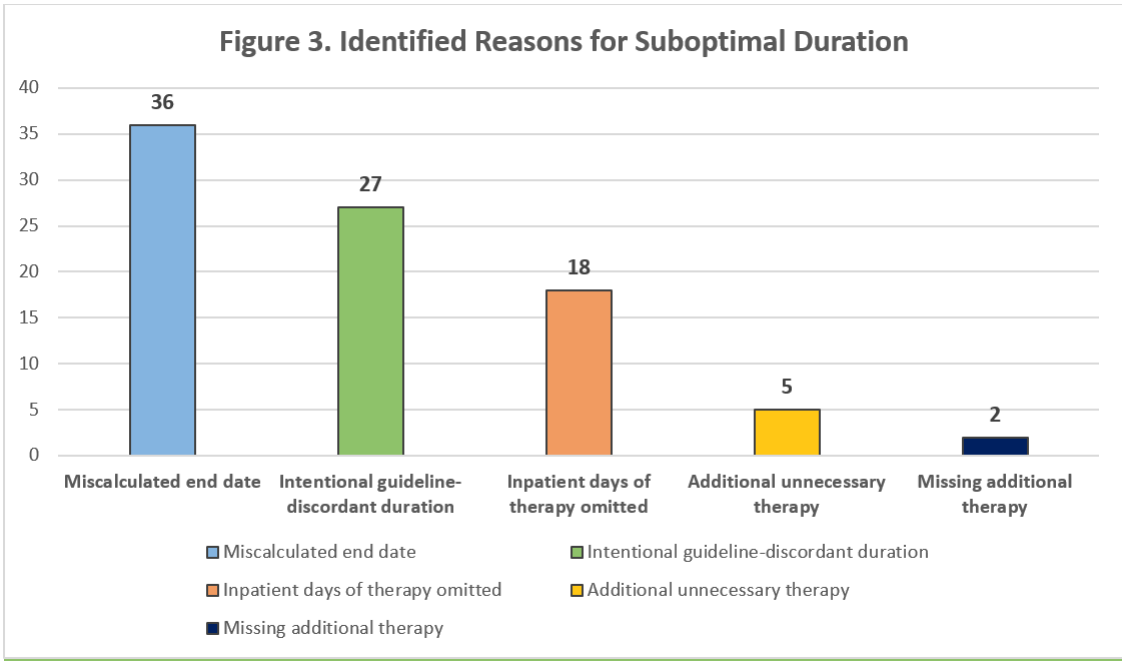

**Conclusion:**

Suboptimal discharge OAPs were common for all studied syndromes, most notably SSTI. Excessive duration was a key driver, with reasons for inappropriate duration previously undescribed. Duration miscalculation and selection of appropriate treatment duration are key areas to focus electronic health record enhancements, provider education, and antimicrobial stewardship efforts.

**Disclosures:**

**All Authors**: No reported disclosures

